# Three-year-olds’ theories of mind in actions and words

**DOI:** 10.3389/fpsyg.2014.00263

**Published:** 2014-03-26

**Authors:** Marjorie Rhodes, Amanda C. Brandone

**Affiliations:** ^1^Department of Psychology, New York UniversityNew York, NY, USA; ^2^Department of Psychology, Lehigh UniversityBethlehem, PA, USA

**Keywords:** theory of mind, social cognition, implicit knowledge, cognitive development, conceptual development

## Abstract

Understanding observable behavior by considering mental representations is central to social cognition. Research reveals quite different developmental trajectories for this ability depending on whether tasks assess implicit or explicit theory of mind (ToM). Yet, how to define implicit vs. explicit ToM, the tasks that elicit each, and the types of behavior that each can support, have remained unclear. The present study (*n* = 47) found that 3-year-olds incorporate predictions based on false beliefs into their intentional actions, but not – following identical scenarios – into their verbal responses. These data show that implicit ToM supports a broader range of behaviors than previously indicated and further illustrates the entrenched nature of the distinction between implicit and explicit knowledge in early conceptual development.

## INTRODUCTION

The ability to understand observable behavior by considering agents’ mental representations of the world is central to human social cognition. This ability is often assessed through false belief tasks, which examine how people expect agents to behave when they have representations that are inconsistent with reality. False belief tasks distinguish whether children predict behavior by considering mental representations or by considering the real state of the world.

On a typical task (Wimmer and Perner, 1983; Baron-Cohen et al., 1985), participants are told that a character, Sally, leaves a marble in her basket and goes outside to play. While she is gone, another character, Anne, moves the marble from the basket to a box. The question posed to participants is some variant of: “When Sally returns, where will she look for her marble?” Or, “Where will she think her marble is?” If participants understand that Sally holds a false belief about the marble’s location and expect her to act on this false belief, they should expect her to look where she left it (i.e., in the basket). If, however, they do not consider Sally’s beliefs, they should base their prediction on her desire and their own knowledge of reality (i.e., she will look in the box because she wants her marble and that is where it is).

For decades, research converged on an intriguing developmental pattern: children age 3 and younger reliably fail these tasks (expecting agents to behave according to their desires or reality, without consideration of representations), then perform at chance (predicting behavior consistent with reality and representation equally often), and finally by age 5, reliably pass these tasks (expecting agents to behave consistent with false beliefs; Wellman et al., 2001). Similar developmental patterns are seen in how children of these ages explain human behavior (Hickling and Wellman, 2001; Amsterlaw and Wellman, 2006). Further, children go through a series of ordered conceptual achievements on route to false belief understanding – understanding first that people act on their own unique desires, then that people act on unique beliefs, next that people only have accurate knowledge if they have had perceptual access to it, and finally that people whose perceptual access has led them astray can hold beliefs that are false (Wellman and Liu, 2004). Thus, the development of theory of mind (ToM) has been described as a process of intuitive theory revision – children first hold simplified theories of human behavior; they then accumulate evidence (e.g., observations of human actions) that is and is not consistent with their theories, leading them to replace earlier theories with more sophisticated understandings (Gopnik and Wellman, 1994, 2012).

These developmental patterns have been the subject of renewed consideration in recent years, as evidence has accumulated that despite the protracted developmental trajectory described above, younger preschoolers and even infants show understanding of false beliefs on more implicit or indirect measures. For example, in nonverbal violation-of-expectation paradigms, after watching events similar to those in the Sally–Anne task, infants as young as 13 months look longer if agents act in line with reality when they ought to hold false beliefs (Onishi and Baillargeon, 2005; Surian et al., 2007; Song and Baillargeon, 2008; Song et al., 2008; Scott and Baillargeon, 2009; Scott et al., 2010). These findings have been interpreted as indicating that infants track the mental representations of agents and expect them to act on false beliefs. Similar results have been found in anticipatory looking experiments; children 18 months and older make predictive gaze shifts indicating that they expect agents to reappear in locations that are sensible only if the agents act on false beliefs (Clements and Perner, 1994; Garnham and Ruffman, 2001; Ruffman et al., 2001; Southgate et al., 2007; Senju et al., 2011; He et al., 2012; Scott et al., 2012).

Perhaps most intriguingly, in the second year of life (ages 18–24 months), infants interact with social partners in manners indicating that they anticipate those partners’ false beliefs. For example, Knudsen and Liszkowski (2012a, b) found that infants spontaneously intervene to prevent people from acting on false beliefs. In particular, infants pointed to the correct location of an object, before their partner committed a mistake. They did not do so when the partner knew the correct location of the object, indicating that infants were able to (a) infer that the person held a false belief, (b) predict how he would behave given that false belief, and (c) spontaneously help their partner by preventing their mistake. Buttelmann et al. (2009) reported that toddlers engage in similar inferences to help their social partners. In particular, they found that, upon seeing a social partner attempt to obtain a desired object from an incorrect location, toddlers helped the partner find the object if the actor was missing during the change of location (and thus could be assumed to hold a false belief), but not if he had been present.

How might this recent evidence of infant’s ToM abilities be reconciled with the protracted developmental trajectory in preschool-age children described above? Some have argued that the infant tasks do not provide evidence of false belief understanding; instead, infants pass these tasks by using a more simplistic ToM concept or applying a set of behavioral rules (Ruffman and Perner, 2005). Yet, as the number of studies reporting positive effects in infants and toddlers using diverse methods has grown, the potential of this explanation to account for the full scope of these new data has diminished.

Alternately, some have suggested that the pattern found in preschooler’s responses does not reflect true conceptual change, proposing instead that infants hold a fully representational ToM, and that observed developments in the preschool years reflect the development of inhibition, language, or executive functioning (Leslie et al., 2004; Baillargeon et al., 2010). This explanation does not account for the full scope of available preschool data, however, for several reasons. First, although executive functioning skills correlate with false belief understanding (e.g., Moses et al., 2005), these variables do not explain all of the variance associated with this conceptual achievement (Wellman et al., 2001, 2011). Second, this perspective cannot account for process-level data on how false belief understanding develops. If the ability to pass classic false belief tasks depended solely on the development of language or executive functioning, it is unclear why children would go through a systematic series of domain-specific conceptual achievements on route to developing false belief understanding (evident on tasks matched for processing demands; Wellman and Liu, 2004; Wellman et al., 2011), or why interventions designed to facilitate the process of theory-change would speed up this development (Amsterlaw and Wellman, 2006; Rhodes and Wellman, 2013). Third, this interpretation cannot account for either the predictors or implications of individual differences in preschooler’s false belief understanding. For example, children who hear more talk about mental states pass false belief tasks earlier (e.g., Dunn et al., 1991), as do children with siblings (e.g., Perner et al., 1994). Passing these classic tasks also uniquely predicts children’s social functioning, including measures of teacher-rated social competence (Watson et al., 1999; Astington, 2003; Peterson et al., 2007) and peer interactions (Dunn et al., 2000; Suway et al., 2012). Thus, despite infants’ nascent abilities, the development of a verbal understanding of false beliefs in preschool is an important, influential conceptual achievement.

A promising approach to reconciling these findings is to accept both as revealing something intriguing about social cognition. Specifically, these two sets of findings may reflect different components of social cognition – implicit and explicit ToM (Apperly and Butterfill, 2009; Low and Perner, 2012). On this account, infants have some implicit knowledge of false beliefs and their role in determining behavior, but this knowledge is not fully accessible to children’s conscious awareness. A fully conscious, explicit ToM must still develop across early childhood through a protracted process of conceptual development. From this perspective, both types of knowledge might contribute to the development of social cognition and to social behavior more generally.

The proposal that children have separate, partially independent, bodies of implicit and explicit knowledge for social cognition should not be surprising. Implicit knowledge operates outside of conscious awareness in many domains, including in numerical cognition, (e.g., Broaders et al., 2007), physical reasoning (e.g., Hood et al., 2003; Hood, 2004), and so on (Apperly and Butterfill, 2009). Also, implicit knowledge commonly precedes explicit knowledge across development (e.g., Siegler and Stern, 1998; Hood et al., 2003). Thus, the two sets of findings described above – documenting discrepancies in children’s abilities depending on whether tasks tap implicit or explicit ToM – are consistent with large literatures in other conceptual domains.

From this perspective, key theoretical questions become how exactly to define implicit vs. explicit ToM, the implications of each for social cognition and behavior, and if and how these theories relate to one another across development. Identifying the circumstances that elicit implicit vs. explicit ToM, and the types of behaviors that each can support, can help to answer these difficult questions. Yet, progress on these fronts has been slow because relatively few studies have directly compared implicit and explicit measures in the same population with the same task (for exceptions, see Clements and Perner, 1994; Garnham and Perner, 2001; Garnham and Ruffman, 2001; Ruffman et al., 2001), making it difficult to determine whether various tasks actually require the same ToM computations and the extent to which differences across studies are attributable to non-theoretically central task demands. Also, few studies have tried to push the boundaries to test the types of behaviors that implicit and explicit ToM can support.

There have been several different proposals regarding how to define implicit ToM. Common themes are that implicit ToM knowledge cannot be articulated and is not incorporated into deliberative judgment (Clements and Perner, 1994; Apperly and Butterfill, 2009), even on an unconscious level (Ruffman et al., 2001). What types of behaviors might such knowledge support? The vast majority of studies revealing evidence of early false belief reasoning have examined looking behaviors, as reviewed above; thus, eye movements appear to reflect implicit ToM. Further, there is clear evidence of a dissociation between eye movements (e.g., anticipatory looks) and verbal responses on standard false belief tasks (Clements and Perner, 1994), suggesting that eye movements and explicit, verbal responses indeed depend on different conceptual systems, even when task demands are equated.

Intriguing additional questions are the extent to which implicit ToM is also capable of supporting intentional action and whether there is a dissociation between intentional action and verbal responses on standard false belief tasks. Vierkant (2012) distinguishes two forms of implicit knowledge, “unaware” implicit false belief understanding, which is encapsulated and does not influence intentional behavior, and “aware” implicit false belief understanding, which can guide intentional behavior, but not children’s deliberative reports. Several recent studies suggest that by 17–24 months, infants’ ToM can support intentional action (Garnham and Perner, 2001; Buttelmann et al., 2009; Southgate et al., 2010; Knudsen and Liszkowski, 2012a, b; see also ; Rubio-Fernandez and Geurts, 2013). Yet few studies have directly compared children’s intentional actions and verbal responses within the same experimental paradigm. Also, most of the paradigms that have been used thus far to examine “ToM in action” have differed markedly from the standard false belief task (see Garnham and Perner, 2001 for an exception). Although these new tasks have many benefits (e.g., providing an assessment of how ToM is actually used; Liszkowski, 2013), it is nevertheless useful to compare action and verbal prediction directly, particularly on tasks that are equated for experimental demands. Further, to better connect the more recent findings to the last several decades of ToM research, it is useful to compare action and verbal prediction in paradigms that are more similar to standard false belief tasks. Doing so was the goal of the present research.

In the current study, we aimed to clarify the types of behaviors that implicit and explicit ToM can support and how each develops across childhood by directly comparing children’s false belief performance across two response modalities: action and verbal responses. The scenarios we used to present characters with false beliefs were identical for both the action and verbal tasks in order that action and verbal responses to these scenarios would be subject to similar inhibition demands (e.g., children must resist acting based on reality and instead act based on beliefs, just like they must inhibit verbally responding based on reality to respond based on beliefs), similar memory demands, and so on. Thus, if children’s actions reflect different knowledge than their words, this would provide clear evidence of the distinction between their implicit and explicit theories in early social cognition. To provide a stringent test of this distinction, we assessed both children’s explicit verbal responses and actions within the very same trial. If children show disparate responses across these two measures even under these circumstances, this would provide strong evidence for an entrenched distinction between implicit and explicit ToM.

## MATERIALS AND METHODS

### PARTICIPANTS

Forty-seven 3-year-olds’ participated (18 male, 29 female; *M* age = 3.60 years, range = 3.03–3.99 years; 52% Caucasian/White; 5% Black/African American; 1% Latino/Hispanic; 9% Asian/Asian American; 33% Multiethnic). By random assignment, 21 children were designated to a true belief (TB) condition; 26 to a false belief (FB) condition. Ten additional children were excluded for refusing to respond (5), difficulty understanding the procedure as evidenced by failing warm-up trials (4), or parental interference (1). Participants were recruited from a database of families who volunteered to participate in developmental research. All parents provided written informed consent and all children provided verbal assent. All procedures used in this research were approved by the University Committee on Activities Involving Human Subjects at New York University.

### PROCEDURE

**FIGURE 1:**
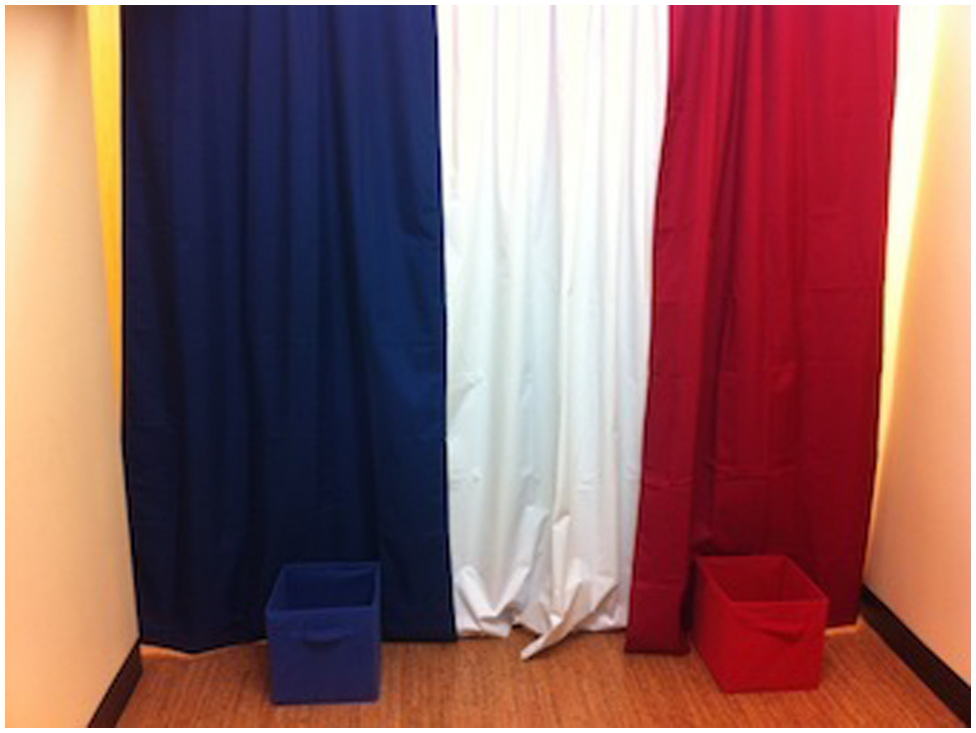
**Photograph of the room set-up for the experimental task**.

Children were brought into a testing room in a campus laboratory. The room was divided into sections by three panel curtains (blue, white, and red) on one wall. A box of the corresponding color was placed in front of the blue and red panels (see **Figure 1**). The child sat in a chair facing the curtains and boxes. The chair was equidistant from the two boxes and curtains. Experimenter 2 (E2) sat in a chair in front of the white curtain, facing the child. When Experimenter 1 (E1) was in the room, she sat on the floor between E2 and the child. The red and blue curtains were indicated to children and referred to as doors (e.g., “See the red door? See the blue door?”).

### WARM-UP

Children completed two warm-up trials. To begin, E1 explained, “I’m going to play with you and some toys. But, I also have to work on some important papers. So sometimes I’m going to go in the back. Then, when it’s time for me to come back in, I’m going to ring the doorbell, and your job is to open the door for me, OK? Let’s all play with this truck.” E2 and E1 then played with the child for approximately 10 s. After the play period, E1 continued, “Now, I need to go work on my papers. I’m going to leave this truck in the blue box. Now, when I want to come back in, I’m going to come through the blue door, which goes with the blue box where the truck is, so I can get the truck again. Remember, I’ll ring the doorbell when I’m ready to come in and you open the door for me as quick as you can.”

Experiment 1 then exited through the middle white curtain using the opening on the side of the blue door (the door where she had left the truck). While E1 was gone, E2 reminded the child, “Remember, your job is to open the door for [E1] when she rings that bell. Where did she leave the truck? So which door should you open for her? OK⋯Let’s wait for [E1].” Across both warm-up trials, children responded accurately on 93% of these questions. If children responded incorrectly, the events were re-described for the child and the questions were re-asked. After 25 s, E1 rang a doorbell (which sounded from a central location), and E2 prompted the child to open the door by saying, “Where’s [E1]?” The child then opened a door for E1 and E1 retrieved the truck from the blue box. If children initially went to the wrong door, they were permitted to try again to find E1 behind the correct door. If the child was hesitant to go to a door, E2 prompted further (e.g., “go ahead and open the door!”), and, if necessary, showed the child how to open the door. The experimenters then played with the child again for 15 s, and completed a second warm-up trial. The second warm-up was identical to the first, except that E1 left the truck in the other box, and thus re-entered through the other door. Where E1 left the truck on the first trial was counter-balanced across participants. Four children failed to open the correct door on any warm-up trial (even after prompting) and were excluded from further analyses. All other children had no trouble understanding the procedure and successfully opened the door on the second warm-up trial. Overall, children opened the correct door on 93% of warm-up trials, with most children (over 80%) opening the correct door on both trials. These accuracy data indicate that children found the basic task straightforward and sensible. The warm-up period thus may have helped children feel comfortable and become familiar with the testing room set-up, but does not appear to have provided a training experience. If the warm-up activities provided training, we would expect to see errors decrease across the two warm-up trials. Instead, overall accuracy rates were very high, and the errors appeared to be randomly distributed. Indeed, performance during the warm-up phase did not relate to performance on any response measure during the subsequent TB or FB trials.

### Experimental Trials

After the warm-up trials, E1 introduced a new toy (a caterpillar). After playing with the new toy briefly, the experimenter announced, “I have to go work on my papers again. I’ll leave the caterpillar in the red box, and I’ll ring the bell when I’m ready to come back in.” E1 then exited through the white curtain using the opening next to the red door.

#### False belief

In the FB condition, after E1 exited, E2 said to the child, “Let’s play a TRICK on [E1]! Let’s be really sneaky and move the caterpillar. She can’t see us. Let’s move the caterpillar from the red box to the blue box! (E2 and the child then moved the caterpillar together.) She can’t see what we’re doing!” To assess children’s comprehension of the key events, E2 then asked a series of questions: “Where did [E1] leave the caterpillar? And where is it now? When we moved the caterpillar, was she watching us?” Children responded correctly, either verbally or by pointing, on 79% of these comprehension questions without further prompting. The majority of errors were on the first question: “Where did [E1] leave the caterpillar?” If children responded incorrectly to this question, the experimenter repeated the question, emphasizing the name of the experimenter so that children realized the question was about the initial location of the caterpillar. If children responded incorrectly to either of the other two questions, the experimenter reminded the child of the events and re-asked the question. No child responded incorrectly a second time to these comprehension questions.

After E1 was outside of the room for 45 s, E1 rang the doorbell, and E2 said, “Where’s [E1]?” The key response was whether children opened the door leading to the box where E1 originally left the toy (FB-based prediction, scored “1”) or leading to the real location of the toy (reality-based prediction, scored “0”). If children did not respond right away, E2 prompted by saying, “Go ahead and open the door for [E1]!” When E1 entered the room, she did not retrieve the object; instead, she simply greeted the child and the trial ended. When it was time to begin the next trial E2 retrieved the object and handed it back to E1.

Children completed two full FB trials. E1 put the toy in the red box on one trial and in the blue box on the other; which box was used first was counter-balanced across participants. To directly compare children’s actions to their verbal responses, on one of the two trials, after the comprehension questions but before the doorbell rang, the child was asked an explicit belief question: “where does [E1] think the caterpillar is?” (scored “1” for a FB-based response, “0” for a reality-based response). Children could respond by pointing to one of the two locations or verbally (e.g., “In the red box.”). To identify whether this question affected children’s subsequent actions, it was asked on only one of the two trials (with order counter-balanced across participants).

#### True belief

The procedures in the TB condition were identical, except that the location change took place before E1 left the room. After E1 stated that she was going to leave, E2 said “before [E1] leaves, let’s play a game with her! [E1], watch this! We are going to move the caterpillar from the red box to the blue box.” E2 and the child then moved the toy together. E1 then exited through the white curtain, on the side of the door where she initially left the toy. The rest of the procedure (including the comprehension questions and the explicit belief question) was identical; similar to the FB condition, children responded accurately on 68% of the comprehension questions without further prompting. As in the FB condition, the majority of errors were on the first comprehension question. All procedures for re-asking the questions were identical to the FB condition. For TB, selections of the door leading to the box where E1 originally left the toy were scored “0,” whereas those leading to the real location of the toy were scored “1,” For the explicit belief question, answers that beliefs would match reality were scored “1.”

It is important to note that on every trial in both FB and TB conditions, E1 exited through the white curtain on the side of the box where she left the toy initially. Thus, a strategy of going to the door closest to where E1 exited would not be a successful strategy, as this would lead to incorrect responses for TB.

## RESULTS

Data were analyzed via binomial regression models. These analyses yield Wald χ^2^ values as indicators of main effects and interactions and odds ratios as indicators of effect size. Data are reported as the probability of giving a belief-based response, accompanied by Wald 95% confidence intervals (CIs). To obtain *p* values for comparisons to chance, we ran null models separately by condition comparing the obtained distribution to that expected by chance.

**FIGURE 2:**
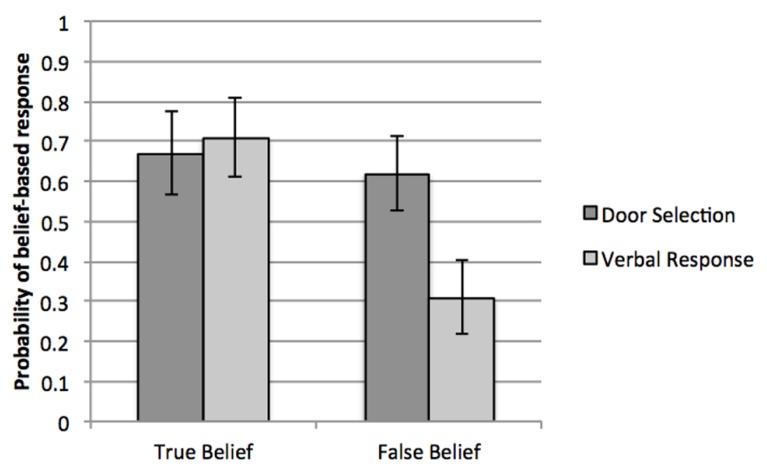
**Probabilities of responses consistent with the actor’s beliefs by response measure, for the single trial per child where they completed both measures**.

To examine the key question of whether there is a distinction between children’s explicit verbal vs. action responses within the very same trial, we tested for the main and interactive effects of condition (TB, FB) and response-type (door selection, explicit question) on the probability of giving a belief-based response to each measure during the trials for which children were asked both the explicit belief question and to open a door. The interaction between condition and response-type was reliable, Wald χ^2^ (1) = 4.83, *p* = 0.03 (see **Figure 2**). In the FB condition, children were more likely to go to the correct door than to answer the explicit belief question correctly (*OR* = 3.60, *CI* = 1.43, 9.05, *p* = 0.006), whereas in the TB condition, children were equivalently accurate on the explicit belief question and the door selection, *p* > 0.60. Also, for the explicit belief question, children responded more accurately in the TB condition than the FB condition (*OR* = 5.63, *CI* = 1.59, 19.85 *p* = 0.007), whereas for the door selections, accuracy did not vary by condition, *p* > 0.70. We also examined individual response patterns on these trials. In the FB condition, children were more likely to pass the door selection but fail the explicit belief question (27% of participants) than to show the reverse pattern (8% of participants), Fisher’s exact test, *p* = 0.02. The remainder of participants either failed both trials (31%) or passed both trials (34%).

To examine children’s door selections more fully, and to test whether children’s door selections were influenced by whether the verbal question was asked prior to the door selection prompt or not, we conducted a follow-up analysis. Using children’s door selections on both trials, we tested for effects of condition (TB, FB) and whether the explicit verbal question was asked prior to the door selection or not. Overall, children selected the door that matched the actor’s beliefs about where the object was located significantly more often than expected by chance (*M* = 0.70, *CI* = 0.58, 0.79, *p* < 0.001). They did so in both the TB (*M* = 0.72, *CI* = 0.56, 0.84, *p* = 0.009) and FB (*M* = 0.68, *CI* = 0.51, 0.81, *p* = 0.04) conditions, with no main or interactive effect of condition, *p* > 0.70. Thus, children were able to use both true and false beliefs to guide their actions. There were no main or interactive effects of whether the explicit belief question was asked prior to the door selection, *p*s > 0.20, suggesting that answering the explicit belief question did not influence children’s subsequent actions. Further, the likelihood of children selecting the correct door did not change across the two trials that each child completed, in either the TB or FB condition, all *p*s > 0.30.

Finally, we examined children’s responses to the verbal question alone, testing for effects of condition (TB, FB) and whether the verbal question was asked on the first or second trial. Consistent with the analysis above, children were significantly more likely to give a belief-based response in the TB condition (*M* = 0.71, *CI* = 0.48, 0.87; comparison to chance, *p* < 0.06) than in the FB condition (*M* = 0.30, *CI* = 0.16, 0.50; comparison to chance, *p* < 0.06), Wald χ^2^ (1) = 6.86, *p* = 0.009, *OR* = 5.63, *CI* = 1.594, 19.85). Thus, in contrast to our findings for the door selection measure, children were better able to use knowledge of beliefs to guide their explicit verbal responses in the TB than FB condition. There were no main or interactive effects of whether the verbal question was asked on participants’ first or second trial, *p*s > 0.50.

## DISCUSSION

In this study, three-year-olds’ used false belief knowledge to guide their intentional actions but not their verbal responses. These findings extend prior work in three key ways. First, they show that, at age 3 (an age when children notoriously have difficulty with standard false belief tasks), children make predictions based on false beliefs to guide intentional actions on tasks that are very similar to the tasks used in classic false belief research. Second, these findings show that even following identical presentation of the events leading to the false beliefs (and thus placing similar demands on inhibition, memory, etc.), the same 3-year-old’s still fail to incorporate false beliefs into their verbal responses. Indeed, the failure rate obtained on our verbal measure was very similar to that reported among this age group in prior work using standard measures of false belief understanding (Wellman et al., 2001). Thus, these data show that, in classic change-of-location tasks, the same 3-year-olds’ can use an understanding of false beliefs to guide their actions, but not their words. Finally, an important feature of our design, which distinguishes this study from prior work (e.g., Garnham and Perner, 2001), is that children completed both verbal and action-based measures within a single trial. Immediately after using their explicit ToM to answer a verbal question (unsuccessfully), children were still able to use their implicit system to guide their actions (successfully). That children responded differently to these measures under these circumstances indicates that the distinction between implicit and explicit ToM is quite entrenched at this age. These data illustrate the firm distinction between implicit and explicit ToM (Apperly and Butterfill, 2009; Low, 2010), and are consistent with prior evidence indicating that the development of an articulated, explicit ToM is the result of a protracted developmental process (Wellman et al., 2001).

A possible issue with examining both action and verbal responses in the same children within a single paradigm is that their responses to one measure might influence their responses to the other for purely methodological reasons. To rule out the possibility that children responded to these measures differently because of some methodological concern (e.g., perhaps children had a bias to change their answers across questions), we also included trials in which children completed only the action measure, without first answering the verbal question. The order of these two trials was counterbalanced across participants. Yet, children responded similarly to the action measure regardless of whether the explicit belief question was asked first (and there were no effects of whether the trial containing the verbal question was asked first or second, *p*s > 0.40). Further, that we found this change in response across measures in the false belief condition, but not the true belief condition, suggests that children were also not subject to a general bias to change their answers across questions. Thus, the pattern that we obtained appears to stem from differences in the conceptual apparatus that supports children’s actions vs. their verbal responses as opposed to from an artifact of our design.

In addition to helping to address the methodological issue described above, the true belief condition also helps to rule out several other possible alternative interpretations of our data. For example, this condition indicates that children understood the direct verbal question, and did not have difficulty answering it simply because the warm-up did not include questions about thoughts. The finding that children performed similarly on the action measure and verbal question in the true belief condition illustrates that the warm-up activities did not inadvertently lead the action-task to be easier to perform than the verbal task. Further, the true belief condition rules out the possibility that children responded by using a simple behavioral rule such as “go to the door from which the actor exited,” or “go to the door by which the actor initially left the object.” Instead, our data support the view that children were using their implicit and explicit ToM to guide their action and verbal responses, respectively.

Baillargeon et al. (2010) proposed that whether young children pass tasks involving false beliefs depends on the response-type of the task: children succeed on measures of spontaneous, non-elicited behaviors, but fail on elicited response tasks. Whether the present data are consistent with this distinction depends on the definition of a spontaneous, non-elicited response task. In the present study, children were told that they were expected to respond and were prompted with the question, “Where’s [E1]?” Thus, this task is not strictly a measure of their spontaneous behavior. Yet, the measure on which children succeeded did not involve a direct question about beliefs; thus, it is possible that they used their false belief knowledge in a spontaneous manner. In future work, it will be useful to directly pit the modality of response (actions, looking, verbal) against task-type (non-elicited vs. elicited) to fully resolve which factor determines whether children rely on explicit or implicit knowledge. For example, it would be useful to examine verbal responses to an explicit question such as, “Which door will the experimenter come through?” This question – though verbal and explicit – could allow children to access their false belief knowledge in a spontaneous manner. Examining responses to this question in future work would allow us to resolve whether the explicit system is activated for any verbal response, or only when children are specifically asked to explicitly consider thoughts.

Perhaps the most important unresolved issue emerging from this work is the relation between implicit and explicit ToM across development. Our data suggest that at least at age 3, the implicit and explicit ToM systems are distinct. Do these systems remain distinct across development, or does early implicit knowledge feed into the development of explicit ToM? Infants’ performance on implicit measures of social cognition correlates with their later false belief understanding, suggesting that these bodies of knowledge are indeed related in some manner (e.g., Wellman et al., 2004, 2008; Thoermer et al., 2012). However, the precise nature of their relation remains unclear. One mechanism we favor is that individual differences in implicit ToM result in differences in the input that children receive to their explicit theory-building system. On this account, infants with more robust implicit ToM become more interested in and attentive to human behavior. Thus, over time, they accumulate more evidence of how human behaviors match or fail to match their explicit ToM, speeding up the process of intuitive theory revision that underlies conceptual development. Further research is needed, however, to examine whether and how implicit ToM varies across individuals and the extent to which this variability predicts children’s social behavior and the development of their explicit theories.

## AUTHOR CONTRIBUTIONS

Marjorie Rhodes and Amanda C. Brandone designed the study, analyzed the data, and prepared the manuscript. Marjorie Rhodes supervised data collection.

## Conflict of Interest Statement

The authors declare that the research was conducted in the absence of any commercial or financial relationships that could be construed as a potential conflict of interest.
